# Quality of life experienced by South Sudanese lower limb prosthetic users after rehabilitation

**DOI:** 10.4102/ajod.v14i0.1671

**Published:** 2025-07-16

**Authors:** Lotto Charles Paul Dominsiano, Surona Visagie

**Affiliations:** 1 Division of Disability and Rehabilitation Studies, Faculty of Medicine and Health Sciences, Stellenbosch University, Cape Town, South Africa

**Keywords:** amputation, health status, quality of life, physical, psychological, social, environmental, prostheses

## Abstract

**Background:**

Prosthetic rehabilitation modifies functional limitations and psychological challenges caused by amputations, as it helps to restore mobility and body image. A physical rehabilitation centre in Juba, South Sudan, has been providing prosthetic rehabilitation since 2009 in this conflict torn part of the world.

**Objectives:**

To determine the quality of life (QoL) of persons with unilateral transfemoral or transtibial amputations who have received prosthetic rehabilitation in Juba, South Sudan.

**Method:**

A cross-sectional survey was conducted with 40 participants, identified through consecutive sampling. Data were collected through face-to-face interviews using the World Health Organization Quality of Life Brief Version (WHOQOL-BREF)questionnaire. Descriptive analysis was conducted, and relationships between QoL and participants’ demographic and medical information were explored through the *t*-test and analysis of variance (normally distributed data) and the Mann–Whitney *U* and Kruskal–Wallace tests (skewed data).

**Results:**

Mean domain scores were physical (74.28%), psychological (72.59%), social (71.40%) and environmental (58.81%). Overall QoL and overall health satisfaction had mean scores of 4.1/5 and 3.975/5. Marital status (0.049) and occupation (0.022) played a significant role in psychological QoL. No other demographic or medical variable had a significant effect on overall or domain QoL scores. Women were significantly more satisfied with their health than men (0.046).

**Conclusion:**

Overall, participants had high QoL scores. This might be because of prosthetic rehabilitation. Lower scores in the environmental domain might be because of poverty and the continuous danger of armed conflict.

**Contribution:**

Even when using basic components, prosthetic rehabilitation can significantly improve QoL after lower limb amputation.

## Introduction

Quality of life (QoL) is defined as an individual’s perception of their position in life and perceived well-being in the context of the culture and value system in which they live and in relation to their goals, expectations, standards and concerns (Calabrese et al. 2023; Deans, McFadyen & Rowe 2008). General QoL is multifaceted and encompasses physical aspects such as pain, mobility and selfcare, psychological aspects such as anxiety and depression, social aspects such as relationships and environmental aspects such as access to services and overarching concepts such as life roles (Ernstsson et al. 2022). Health-related QoL (HQoL) focuses specifically on health properties (Calabrese et al. 2023). The focus of this study was in general QoL.

As summarised by Calabrese et al. (2023) in a recent literature review, an amputation has a negative effect on QoL. These authors further found that QoL improves over time after the amputation (Calabrese et al. 2023). However, it is unclear whether the improvement reaches pre-amputation levels of QoL. Studies from Asia and Africa showed that an amputation can have a negative effect on the physical, psychological, social and environmental domains of QoL (Adegoke et al. 2012; Deepak et al. 2023; Nizamli 2020; Razak et al. 2016; Shankar et al. 2020; Zaheer et al. 2020). Physical QoL is often affected most severely as the amputation directly impacts mobility and physical functioning (Adegoke et al. 2012; Deepak et al. 2023; Razak et al. 2016; Shankar et al. 2020; Zaheer et al. 2020). Mean physical QoL scores varied between 43.37 in a study conducted in Syria with 65 men who had lower limb amputations because of war injuries (Nizamli 2020) and 61.6 among 37 Malaysians with lower limb amputations because of various causes (Razak et al. 2016). Conversely, all studies (Adegoke et al. 2012; Deepak et al. 2023; Shankar et al. 2020; Zaheer et al. 2020), except for two (Nizamli 2020; Razak et al. 2016), found social QoL to be the least affected by amputation.

### Variables that influence quality of life after amputation in African studies

Authors agree that more proximal levels of amputation (Calabrese et al. 2023; Migaou et al. 2019) and non-traumatic amputation (Migaou et al. 2019) significantly reduce QoL. The use of a prosthesis (Adegoke et al. 2012; Chunteng et al. 2022; Enweluzo et al. 2023; Hando et al. 2023; Von Kaeppler et al. 2021) and the absence of stump pain or phantom limb pain (Hando et al. 2023) have a positive effect on QoL after amputation.

Regarding demographics, African research showed conflicting findings. Adegoke et al. (2012) showed that male sex had a significant positive influence on physical and social QoL among Nigerians with lower limb amputations. However, also in Nigeria, Enweluzo et al. (2023) found that women had significantly better physical and psychological QoL than men. Chunteng et al. (2022) found that sex did not influence QoL after amputation. According to Hando et al. (2023), tertiary education and employment positively influenced QoL. However, Chunteng et al. (2022) found that education, occupation and income status had no influence on QoL

Quality of life of persons with amputation has been explored in several African settings including parts of Cameroon (Chunteng et al. 2022), Nigeria (Adegoke et al. 2012; Enweluzo et al. 2023), Tanzania (Hando et al. 2023) and Tunisia (Migaou et al. 2019). However, most of these studies focused on HQoL and on determining variables that influence QoL or HQoL. None of them was done in South Sudan, a war-torn country, and none compared the QoL of persons with amputation to the QoL of the general populations.

### Context: South Sudan and Juba rehabilitation centre

It is unknown how many persons live with limb loss in South Sudan. However, according to the International Committee of the Red Cross (ICRC), 1894 persons received lower limb prostheses between July 2022 and July 2023 in South Sudan. Out of this number, 1800 received transtibial prostheses and 94 received transfemoral prostheses. In South Sudan, lower limb amputation is commonly caused by gunshot wounds, landmines, snake and animal bites, road traffic accidents (RTAs) and diabetes (Rohwerder 2018). With a long civil war, the prevalence of lower limb amputation has increased in South Sudan, leading to higher demand for prosthetic services.

**FIGURE 1:**
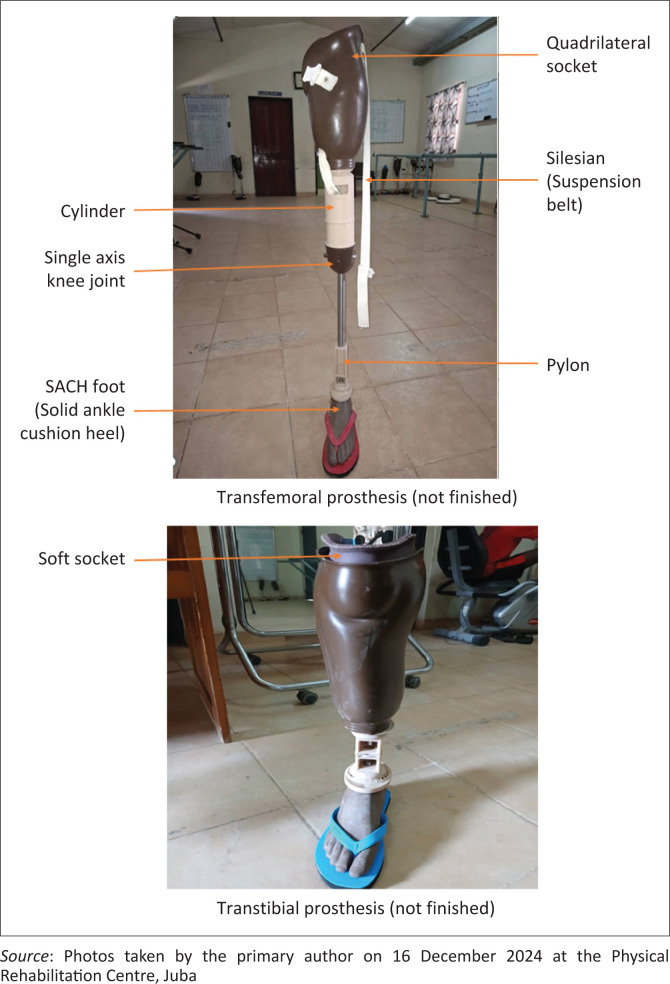
Images of the prostheses and components used.

A physical rehabilitation centre, one of three centres in the country that provide prosthetic rehabilitation, was established in Juba in January 2009 by the government of South Sudan. The aim of the centre is to provide rehabilitation services to wounded soldiers, particularly those who have lost limbs. The centre is supported by the ICRC. Between 20 and 30 persons with transfemoral or transtibial amputation are admitted to the centre per month. Prosthetic components used at the rehabilitation centre are basic in nature. All transfemoral and transtibial prostheses are manufactured with a solid ankle cushion heel (SACH) foot. Transtibial prostheses have a patella tendon bearing, or a patella tendon bearing supra-condylar socket, or, if the stump is very short, a thigh corset. Transfemoral prostheses have a single axis knee, quadrilateral or ischium containment socket and belt and sling suspension (see Figure 1).

No studies focusing on the QoL of persons with lower limb amputations using prostheses have been done at the rehabilitation centre in Juba since its establishment. Without this knowledge, service providers do not know whether their services are effective in improving user QoL. They also do not know which variables increase or decrease QoL. This knowledge might help to optimise services and thus improve QoL of future users. Thus, this study aimed to determine the QoL of persons with unilateral transfemoral or transtibial amputations who have received prosthetic rehabilitation in Juba, South Sudan.

The research question was, ‘what is the experienced QoL of persons who have received transfemoral or transtibial prosthetic rehabilitation in South Sudan?’ The main study hypothesis stated that the QoL of persons who have received transfemoral or transtibial prosthetic rehabilitation in South Sudan was poor. A secondary hypothesis stated that demographic and amputation-related variables (age, gender, level of education, marital status, occupation, cause of amputation, level of amputation, use of handheld assistive devices and stump length) did not influence QoL. A final hypothesis stated that QoL of persons who have received transfemoral or transtibial prosthetic rehabilitation in South Sudan will be lower than the QoL of general populations.

## Research design and methodology

*Design:* A quantitative, descriptive, cross-sectional design was used to collect data at one point in time over a short time frame (Wang & Chang 2020).

*Population, sampling and recruitment:* The study population included all persons with unilateral lower limb amputations who have received prosthetic rehabilitation at the study setting. The total number was unknown. To be included, persons had to be 18 years or older, amputated either transfemoral or transtibial, have received the prosthesis more than 3 months ago (Zidarov, Swaine & Gauthier-Gagnon 2009 found that amputees performed significantly more activities with their prosthetics at 3 months than at discharge), and have used their prosthesis. Those who have stopped using the prosthesis were excluded as prosthetic use can influence QoL (Wurdeman, Stevens & Campbell 2018).

A total of 40 participants were sampled using consecutive sampling because it controls sampling bias better than other non-probability sampling methods by including all available persons who adhere to the inclusion criteria (Thewes et al. 2018). No sample size calculation was done because the size of the study population was not known. While it was known how many persons received prosthesis per month, there was no way to determine how many would visit for follow-up services during the data collection period. O’Leary (2017) has indicated that a sample of 30 or more is sufficient for the analysis of quantitative data. Similar studies have been done with 37 (Razak et al. 2016), 47 (Adegoke et al. 2012) and 50 (Kalsoom, Amjad & Bairam 2018) participants.

The primary author identified inpatients and outpatients who adhered to the inclusion criteria, and who received prosthetic rehabilitation at the study setting, with the assistance of a social worker. The primary author approached potential participants and shared information about the purpose of the research and their role in it with them. An interpreter assisted where participants were not fluent in English or Arabic (3/40). The primary author read the question, which was then translated to the participant by the interpreter. The participant answered, and the interpreter translated the answer to the primary author.

*Data collection:* Data were collected with the World Health Organization Quality of Life Brief Version (WHOQOL-BREF) questionnaire. The WHOQOL-BREF is a validated reliable (WHO 1998) instrument that has been used widely in population-based studies as well as in populations of persons with lower limb amputations (Calabrese et al. 2023). According to Calabrese et al. (2023), the WHOQOL-BREF is the survey tool most used to explore QoL or HQoL among persons with amputation. Specific tools that focus on measuring QoL in amputee populations, like the orthotic and prosthetic users survey – health QoL (Jarl et al. 2012), and measures that look at HQol, such as the Short Form Health Survey 36 (SF-36), are available. However, the WHOQOL-BREF was used in this study rather than any of the other tools as the focus was on general QoL, not HQoL, and findings were compared to population-based norms.

The WHOQOL-BREF (WHO 1998) was developed by the WHO for cross-cultural comparison of QoL. It can be downloaded for free from https://www.who.int/tools/whoqol/whoqol-bref. It provides a valid, accurate and convenient assessment of QoL and can be completed within a short period of time (WHO 1998). The WHOQOL-BREF questionnaire contains 26 items consisting of four domains:

- Physical health (seven items related to pain, health care needs, energy, mobility, sleep, daily activities and work)
- Psychological health (six items related to life enjoyment, positive attitudes, concentration, body image, self-esteem and negative feelings)
- Social relationship (three items related to personal relationships, sex life and support)
- Environmental health (eight items related to safety, healthy environment, finances, information access, leisure activities, living place, access to health care and transport)
- And two general items, that is, general QoL and general health (WHO 1998).

The score of each individual item ranges from 1 to 5 on a Likert scale. Higher scores denote better QoL.

Questions on cause and level of amputation, length of time having a prosthesis and using additional handheld assistive devices were added to the demographic questions of the WHOQOL-BREF for this study.

The WHOQOL-BREF questionnaire was completed during face-to-face structured interviews. For the three participants who did not understand Arabic or English, the two languages in which the survey was available, the primary author read the questions in English, and an interpreter translated them into a language that the participant was well versed in. It took 15 min – 20 min to complete the questions. Data collection was done between March and June 2024.

*Data management and analysis:* There were no missing data. All data were coded and entered into Excel. In accordance with the WHOQOL-BREF scoring guidelines, the negatively phrased items were reversed before coding. The data were nominal and ordinal categorical, as well as ratio. Nominal data included gender, level of amputation, residual limb length and occupation. Most data were ordinal because survey responses were scored on a 5-point Likert scale. These scores were used to calculate domain scores that were converted to percentages. Domain scores, together with scores on age and length of time using a prosthesis, provided numerical data.

Nominal and ordinal data were summarised in percentages and modes. The QoL item and domain scores were descriptively analysed for central tendency and spread through means and standard deviations.

Calculation of QoL domain scores was done according to the provided guidelines (https://www.who.int/tools/whoqol/whoqol-bref). Domain scores were computed and transferred to a score out of 100 to compare different domains with each other and for comparison with other studies, as per the WHOQOL user manual instructions. The mean score (raw domain score) in each domain was determined by adding the scores of the questions relevant to that domain and dividing it by the number of items in the domain. Mean scores were multiplied by four to make them comparable to WHOQOL-100 scores. They were then transformed to a 0–100 scale by subtracting four from the WHOQoL-100 score and multiplying the answer with 100/16.

Further analysis was done to determine if there were any significant relationships between demographic and medical information, such as age or level of amputation, in relation to overall and domain QoL scores. The Shapiro–Wilk test showed that data for the physical and environmental domains were normally distributed. Thus, inferential analysis was done with the *t*-test to compare the means between two groups (gender, level of amputation and use of handheld devices) and analysis of variance (ANOVA) to compare the means across three or more groups (level of education, marital status, occupation, stump length) for these two domains. Data for the psychological and social domains were not normally distributed, and further analysis was done with the Mann–Whitney *U* test (comparison between two groups) and the Kruskal–Wallace test (comparison between three and more groups). Alpha < 0.05 was deemed statistically significant. Statistical Package for the Social Sciences (SPSS) version 29 was used to analyse data with the support of a statistician from the Stellenbosch University biostatistics centre.

### Ethical considerations

*Approval:* Ethical approval was received from the Stellenbosch University Health Research Ethics Committee (S23/10/256). The Ministry of Health and Research Ethics and Review Board (MOH-RERB A-77/2023) in South Sudan provided permission to do the study in the country. Permission was also obtained from the manager of the rehabilitation centre.

*Respect for persons:* Both verbal and written informed consent were sought. Translators were utilised as the participants were from diverse communities and spoke different languages. No participant withdrew from the study.

*Familiarity with the primary author:* The primary author knew some of the participants. These participants were given the same respect, explanation and opportunity to refuse as those who did not know him. The primary author explained that the prior relationship should not influence their choice to participate or the answers they provided.

*Beneficence and non-maleficence:* The research did not pose a risk of physical harm. No participant experienced emotional distress during data collection. The information gathered was used for research purposes only. Data were kept confidential and only shared with the study supervisor and statistician. Paper copies of completed surveys are stored in a locked cupboard. The electronic spreadsheet copy was saved on a computer and backed up on an external hard disk. Both were locked with a password. The data will be kept for 5 years, after which it will be destroyed. All participants were informed that they would not be paid to participate in the study; however, they received money for transport and tea during the interview.

*Distributive justice:* All participants were treated with respect and dignity, irrespective of ethnic, religious or political affiliation.

## Results

### Demographic details

More males (27) compared to females (13) participated in the study, with a percentage of 67.5 versus 32.5. The mean age was 42.3 years (standard deviation [s.d.] 13.6). The minimum age of the participants was 19, and the maximum was 69. Table 1 shows that 42.5% (17/40) of the participants attended primary school, while 7.5% (3) achieved tertiary education, and 27.5% (11) did not have any formal education. The majority (82.5%, 33) of the participants were married. Most of the participants (65%, 26) were unskilled labourers. Of the 13 women participants, 84.6% (11) were unskilled, and of the 27 males, 55.6% (15) were unskilled. The minimum and maximum years of wearing prosthesis were 0.3 and 32, respectively. Participants had been using their prostheses for a mean period of 13.7 (s.d. 9.5) years.

**TABLE 1:** Distribution of demographic and amputation-related information of the study participants.

Variables	Male		Female		Total	
	*n*	%	*n*	%	*N*	%
**Education level**						
None	5	12.5	6	15.0	11	27.5
Primary	12	30.0	5	12.5	17	42.5
Secondary	7	17.5	2	5.0	9	22.5
Tertiary	3	7.5	0	0.0	3	7.5
**Marital status**						
Married	26	65.0	7	17.5	33	82.5
Never married	1	2.5	3	7.5	4	10.0
Widowed	0	0.0	3	7.5	3	7.5
**Occupation**						
Unskilled	15	37.5	11	27.5	26	65.0
Skilled	5	12.5	1	2.5	6	15.0
Protective services	5	12.5	0	0.0	5	12.5
Student	2	5.0	1	2.5	3	7.5
**Causes of amputation**						
Gunshot	15	37.5	7	17.5	22	55.0
Landmine	5	12.5	2	5.0	7	17.5
Road traffic accident	4	10.0	1	2.5	5	12.5
Infection or vascular	2	5.0	2	5.0	4	10.0
Snake or animal bite	1	2.5	1	2.5	2	5.0
**Level of amputation**						
Transtibial amputation	15	37.5	10	25.0	25	62.5
Transfemoral amputation	12	30.0	3	7.5	15	37.5
**Residual limb length**						
Short	9	22.5	5	12.5	14	35.0
Medium	13	32.5	6	15.0	19	47.5
Long	5	12.5	2	5.0	7	17.5
**Handheld devices**						
No	21	52.5	9	22.5	29	75.0
Yes	6	15.0	4	10.0	11	25.0

### Amputation-related results

Trauma related to gunshot wounds was the major cause of amputation among the participants, accounting for 55.0% (22) and occurred throughout the age groups. This was followed by land mine injuries at 17.5% (7). When RTA and animal bites are added, 85.0% (34) of the amputations were done because of trauma. Transtibial amputation (62.5%, 25) was more common than transfemoral amputation (37.5%, 15). No other level of major lower limb amputation was recorded during the study. In most instances, the residual limb length was medium (47.5%, 19). Most of the participants (75.0 %, 29) did not need additional handheld assistive devices such as crutches or walking frames. Landmine injuries were more common among persons older than 42 years, while vascular causes and animal bites were more common in the younger age groups (Table 2).

**TABLE 2:** Causes of amputation according to age category.

Causes of amputation	18–25 (years)		26–33 (years)		34–41 (years)		42–49 (years)		≥ 50 (years)		Total	
	*n*	%	*n*	%	*n*	%	*n*	%	*n*	%	*N*	%
Gunshot	3	7.5	2	5.0	6	15.0	3	7.5	8	20.0	22	55.0
Landmine	0	0.0	0	0.0	1	2.5	2	5.0	4	10.0	7	17.5
RTA	1	2.5	0	0.0	1	2.5	2	5.0	1	2.5	5	12.5
Infection or vascular	1	2.5	1	2.5	2	5.0	0	0.0	0	0.0	4	10.0
Snake or animal bite	1	2.5	1	2.5	0	0.0	0	0.0	0	0.0	2	5.0

**Total**	**6**	**15.0**	**4**	**10.0**	**10**	**25.0**	**7**	**17.5**	**13**	**32.5**	**40**	**100.0**

RTA, road traffic accident.

### Quality of life results

Three domains, that is, physical (74.28), psychological (72.59) and social (71.40), had a mean score above 70. The environmental domain had a much lower mean score of 58.81. At 75, the social domain showed the widest range of scores. Overall QoL and overall health satisfaction had mean scores of 4.1 and 3.975, respectively (Table 3).

**TABLE 3:** Descriptive analysis of quality of life domain scores.

Statistical descriptor	Physical domain		Psychological domain		Social domain		Environmental domain		Overall QoL	Overall health satisfaction
	Raw (out of 35)	100	(Raw out of 30)	100	Raw (out of 15)	100	Raw (out of 40)	100	Out of 5	Out of 5
Minimum	21.00	50.00	16.00	42.50	5.00	17.00	20.00	37.50	3.00	3.00
Maximum	34.00	97.50	28.00	92.00	14.00	92.00	32.00	75.00	5.00	5.00
Range	16.00	47.50	12.00	50.00	9.00	75.00	12.00	37.50	2.00	2.00
Mean	27.93	74.28	23.40	72.59	11.50	71.40	26.70	58.81	4.10	3.98
S.d.	10.03	10.00	2.180	8.42	1.70	14.40	2.45	7.86	0.55	0.53
Median	28.00	75.00	24.00	75.00	12.00	75.00	27.00	60.00	4.00	4.00

s.d., standard deviations; QoL, quality of life.

Table 4 shows the four questions with the highest and the lowest overall scores. The lowest scoring question, and the only question scoring below 100/200, was about the availability of money to meet needs (76/200). (The highest possible score is 5 when multiplied by total number of participants [40] gives 200).

**TABLE 4:** Descriptive analysis of individual World Health Organization quality of life BREF questions with the highest and lowest overall scores.

Question	Score out of 200
**Highest scoring questions**	
How much do you need any medical treatment to function in your daily life?	175
To what extent do you feel that physical pain prevents you from doing what you need to do?	164
Are you able to accept your bodily appearance?	162
How satisfied are you with your sex life?	161
**Lowest scoring questions**	
How available is the information that you need in your day-to-day life?	138
How healthy is your physical environment?	133
To what extent do you have the opportunity for leisure activities?	128
Have you enough money to meet your needs?	76

### Comparative analysis

Unskilled occupations (0.022) led to significantly lower QoL scores in the psychological domain. Being married (0.049) showed a slight positive impact on psychological QoL. None of the other demographic variables had a significant impact on overall or domain QoL scores. However, women were significantly more satisfied with their health than men (0.046). Five (18.5%) men were not satisfied with their health versus only one (8.3%) woman. Persons with unskilled occupations (0.027) were significantly less satisfied with their health than those in other occupational categories (Table 5).

**TABLE 5:** A comparison between independent variables, quality of life domain, overall quality of life, and health satisfaction scores using the *t*-test, analysis of variance, Mann–Whitney *U* test and Kruskal–Wallace test as described under data analysis.

Demographic	Physical		Psychological		Social		Environmental		QoL	Health
	*P*	*d*/Eta^2^/z/H**	*P*	*d*/Eta^2^/z/H**	*P*	*d*/Eta^2^/z/H**	*P*	*d*/Eta^2^/z/H**	*P*	*P*
Gender	0.088	9.68	0.186	−1.32	0.210	−1.25	0.634	7.94	0.055	0.046*
Level of amputation	0.254	10.02	0.481	−0.70	0.885	−0.14	0.588	7.94	0.859	0.643
Handheld assistive devices	0.517	10.09	0.170	−1.37	0.501	−0.67	0.827	10.9	0.639	1.000
Level of education	0.197	0.12	0.946	0.11	0.075	5.17	0.140	0.41	0.678	0.527
Marital status	0.278	0.07	0.049*	6.04	0.692	0.74	0.488	0.04	0.600	0.102
Cause	0.367	-	0.132	7.08	0.904	-	0.323	1.04	0.647	0.330
Occupation	0.184	0.12	0.022*	-	0.111	-	0.381	0.08	0.806	0.027*
Stump length	0.581	0.03	0.331	2.21	0.114	4.35	0.104	0.12	0.115	0.457

ANOVA, analysis of variance; QoL, quality of life.

* , *P* < 0.05 (statistically significant).

** , Effect sizes as determined by Cohen’s *d* (*T*-Test), Eta square (ANOVA), *z* (Mann–Whitney *U* test) and *H* statistic (Kruskal–Wallace test).

## Discussion

The main study hypothesis was rejected as the QoL of persons who have received transfemoral or transtibial prosthetic rehabilitation in South Sudan was good rather than poor. Quality of life scores were especially high in the physical, psychological and social domains. The environmental domain had a much lower mean score. The secondary hypothesis was accepted except for occupation and marital status, which had a significant impact on psychological domain QoL scores. The final hypothesis was rejected as the QoL of persons who have received transfemoral or transtibial prosthetic rehabilitation in South Sudan was similar to the QoL of general populations.

*Quality of life:* At 74.28, the mean physical domain score was higher than the mean scores of the other three domains. This is in contrast with other studies that found physical domain scores to be lower than scores of other domains (Adegoke et al. 2012; Deepak et al. 2023; Hando et al. 2023; Razak et al. 2016; Shankar et al. 2020; Zaheer et al. 2020), as one might expect from a condition that impacts mobility directly. Most participants reported no pain during physical activities, no need for medical treatment to function in their daily lives, and satisfaction with sleep, work and mobility, all adding to high physical domain scores. The absence of pain or pain management (Banskota et al. 2024; Hando et al. 2023), satisfaction with sleep (Razak et al. 2016), satisfaction with ability to work (Razak et al. 2016) and being mobile (Hando et al. 2023) are important in improving QoL after amputation. Furthermore, not all participants in the previous studies had a prosthesis. This might further explain the difference in scores as prostheses are associated with increased QoL after amputation (Adegoke et al. 2012; Chunteng et al. 2022; Enweluzo et al. 2023; Hando et al. 2023; Von Kaeppler et al. 2021).

The environmental domain mean score was at 58.81 around 15% lower than mean scores for the other three domains. Nizamli (2020) also found the lowest mean score in the environmental domain (39.65) in a study conducted in Syria. Both this study and the study by Nizamli (2020) were conducted in war-torn settings. The continuous conflict, with the presence of soldiers, as well as the risk of skirmishes and landmines, makes the environment unsafe (Rohwerder 2018). Individuals might hesitate to move about their community and perform activities outside the house as they would like to, thus reducing their environmental QoL. The low score may also be related to poor attitudes of the community towards persons with lower limb amputations, poor infrastructure and poor transport systems (Rohwerder 2018). Most participants reported that they could not access the information that they need in their daily lives, the physical environment was not friendly, they did not have much opportunity for leisure time, they were not satisfied with transport and most of them did not have enough money to meet their needs. The financial situation might be because most of the participants did not have secondary or tertiary education; as a result, they struggled to access skilled jobs.

The findings that overall QoL (4.10) and overall satisfaction with health (3.98) scores were good suggest that participants adapted to life with an amputation and prosthesis to the point where it did not have much of an effect on their QoL and health satisfaction. The overall QoL scores were higher than findings by Adegoke et al. (2012) on the QoL of Nigerians with lower limb amputations (3.91) and Nizamli (2020) on the QoL of Syrians with war-related lower limb amputations. The high scores in this study might be related to an active lifestyle and the availability of a well-equipped rehabilitation centre, which provided not only assistive devices but also comprehensive training and psychosocial support through counselling, as rehabilitation has been associated with improved QoL after amputation (Enweluzo et al. 2023). Prosthetic and rehabilitation services were provided free of charge by the government under the Ministry of Gender, Child and Social Welfare with support from the ICRC in South Sudan.

### Influence of demographic and amputation-related variables on quality of life

The ratio of male to female participants was around 2:1. The higher prevalence of amputation among men is common globally (Bernatchez, Mayo & Kayssi 2021), and in Africa (Talona et al. 2016; Tchankoni et al. 2024; Ukibe et al. 2016), but the reasons for this are less clear. In this study, the higher number of men, and the high rates of gunshot and landmine injuries as a cause of amputation, might be because of the long civil war, known as the Any-anya I rebellion from 1955 to 1972, followed by the Any-anya II rebellion from 1983 to January 2005 (Ensor 2012, 2013). Men, including young boys, were recruited into the Sudan People’s Liberation Army, also known as the Red Army (Ensor 2012), while women were left to take care of the home and children. Mlambo, Mpanza and Mlambo (2019), in their study about armed conflict and the increasing use of child soldiers in the Central African Republic, Democratic Republic of Congo and South Sudan, found that child soldiers were common in those countries. Cherwon (2014) also found that Uganda and South Sudan were recruiting young children into the army. This might help to explain the finding that up to 36% of persons with gunshot trauma and 40% with landmine injuries that caused the amputation were among the age group ≥ 50 years, that is, the group that would have been young men or children at the time of the rebellions.

Vascular disease and diabetes mellitus are the leading causes of amputation in large parts of the world (Chalya et al. 2012; Ernstsson et al. 2022; O’Keeffe & Rout 2019), including parts of Africa (Limakatso et al. 2024; Mohammed & Shebl 2014). However, the picture differs in war-torn areas. Over 70% of persons who have received rehabilitation after lower limb amputation from the ICRC in Afghanistan, Cambodia, Iraq, Myanmar, and Sudan suffered an amputation because of trauma, of which 48.6% were conflict-related (Barth et al. 2021). Hawari et al. (2017) also noted that industrial accidents, RTA and war-related injuries are leading causes of amputation in many low-income countries. In conflict situations, amputation is often the only treatment option available because of limited resources and personnel (Shankar et al. 2020), severity of the injury and the presence of ischaemic, infected or necrotic tissue (Razak et al. 2016).

This study also found that most participants were not educated or received little education, particularly females, of whom none achieved tertiary education, and only two had secondary education. A study by Buckinx et al. (2021) found that out of 490 South Sudanese participants, 64% of females had never been to school compared to 38% of males. The gender gap widens when it comes to secondary education. This is partly because primary schools are available in communities, but secondary schools are less common and often far away. Parents are not willing to let their daughter walk long distances to school because of safety concerns and in fear of rape, which may result in an unwanted pregnancy (Oxfam 2017). Furthermore, girls sometimes did not attend school because of early marriage (Oxfam 2017). Culturally, young girls are married off by their families to get the bride price as a source of wealth (Aleu, Ayii & Amos 2024). Aleu et al. (2024) found that child marriage was widely practiced in South Sudan, with 52% of South Sudanese girls getting married before the age of 18. Child marriage is related to poverty and illiteracy. The finding that males did not attend school or only attended primary school might also be because of the conflict in the country with school-aged boys being recruited into the army (Ensor 2012, 2013).

Marital status might have influenced psychological QoL positively because a spouse can provide emotional support, including motivation and encouragement, which can be important to reduce negative feelings, anxiety, depression and loneliness. In addition, a spouse can add to the household income reducing stress about providing for basic needs.

Unskilled occupations were associated with decreased psychological QoL and decreased health status. This might be because unskilled jobs often involve repetitive activities with little or no exposure to different tasks or environments, hence no opportunity for career growth. This can lead to feelings of frustration and stress, which affect the psychological well-being of an individual. Unskilled jobs are low paid, and income might not meet the cost of basic needs. According to Deepak et al. (2023), people with better financial stability have access to high quality services including access to essential basics to cater for themselves and the family.

Women were more satisfied with their health than men; this might be because women usually share their thoughts and feelings more openly than men who might be more reticent. Women might also be more involved in social activities in the community, which can encourage a sense of well-being and reduce feelings of loneliness and frustration. The finding agrees with that of Enweluzo et al. (2013) but is contrary to that of Adegoke et al. (2012), who found that male participants had significantly higher overall health scores (0.012) than their female counterparts.

*QoL of study participants compared to that of general populations:* The mean domain scores found in this study were more comparable with those of the population-based studies presented than studies conducted with persons with amputations. Mean scores for the four domains in population-based studies across Victoria, Australia (Hawthorne, Herrman & Murphy 2006), Kuwait (Ohaeri, Awadalla & Gado 2009), France (Baumann et al. 2010), Brazil (Cruz et al. 2011), Portugal (Patrício et al. 2014), Indonesia, (Purba et al. 2018), South East Australia (West et al. 2023) and Mongolia (Bat-Erdene et al. 2023) are 66.65 (physical domain), 69.28 (psychological domain), 71.39 (social domain) and 67.5 (environmental domain). Thus, this study scores are higher in the physical and psychological domains, similar in the social domain and lower in the environmental domain. The scores might be comparable with normative data because the prosthesis supports function to a point where users can continue with their lives as before the amputation as described by Buetow, Martínez-Martín and McCormack (2019). However, this is an area that must be further researched.

### Strengths, weaknesses and rigour

The primary author previously provided physiotherapy to some of the participants. They might have been hesitant to share negative feelings with him. He assured participants that the information was to gain a better understanding and that sharing negative information would not put them in a poor light with him or influence their relationship with him. The WHOQOL-BREF was tested in 23 countries with diverse cultures and different socioeconomic development levels. It was found valid and reliable in assessing QoL (WHO 1998). However, it was not tested in South Sudan. To further ensure reliability, the primary author ensured that participants understood each question and used an Arabic version where relevant. But some meaning might have been lost where interpreters were used. Not calculating sample size and the low number of participants negatively influence the generalisability of the results. Finally, the length of use of the prostheses was not considered in QoL scores. Living longer with an amputation and longer use of a prosthesis can negate an initial reduction of QoL (Calabrese et al. 2023). Data were collected at one point in time and completed within a short time frame. Questionnaires were carefully checked to make sure that all questions were answered. Consecutive sampling reduced bias as it guided inclusion of all available population members (Thewes et al. 2018).

### Recommendations

*Clinical relevance:* The results revealed good QoL; therefore, the stakeholders who are involved with the rehabilitation of persons with lower limb amputations in the setting should continue their rehabilitation. More attention should be given to the psychological status and overall health of those in unskilled occupations during rehabilitation. It is also important to increase awareness among the government and the general population that aspects of the physical environment must be improved.

*For further research:*

- A prospective study is recommended to determine whether there are changes in QoL between the time of admission, at discharge and after follow-up in the community, which would provide more information on QoL before and after prosthetic provision.
- A cohort study in which the QoL of persons with amputation without prosthesis, persons with amputation with prosthesis and persons without amputation living in the same communities is compared.
- Studies exploring the role of prostheses in normalising function and QoL to a point that is comparable to normative data on QoL.

## Conclusion

This study tells us that participants who have received a transfemoral or transtibial prosthesis at the study centre had good QoL in the physical, psychological and social domains after prosthetic rehabilitation. Although the environmental domain scored lower, QoL in this domain was still moderate. The availability of a well-equipped rehabilitation centre with dedicated staff assisted persons with transfemoral or transtibial amputation to adapt to the prosthesis and life after amputation.
